# MiRduplexSVM: A High-Performing MiRNA-Duplex Prediction and Evaluation Methodology

**DOI:** 10.1371/journal.pone.0126151

**Published:** 2015-05-11

**Authors:** Nestoras Karathanasis, Ioannis Tsamardinos, Panayiota Poirazi

**Affiliations:** 1 Department of Biology, University of Crete, Heraklion, Greece; 2 Institute of Molecular Biology and Biotechnology (IMBB), Foundation of Research and Technology Hellas (FORTH), Heraklion, Greece; 3 Department of Computer Science, University of Crete, Heraklion, Greece; 4 Institute of Computer Science (ICS), Foundation of Research and Technology Hellas (FORTH), Heraklion, Greece; Roswell Park Cancer Institute, UNITED STATES

## Abstract

We address the problem of predicting the position of a miRNA duplex on a microRNA hairpin via the development and application of a novel SVM-based methodology. Our method combines a unique problem representation and an unbiased optimization protocol to learn from mirBase19.0 an accurate predictive model, termed MiRduplexSVM. This is the first model that provides precise information about all four ends of the miRNA duplex. We show that (a) our method outperforms four state-of-the-art tools, namely MaturePred, MiRPara, MatureBayes, MiRdup as well as a Simple Geometric Locator when applied on the same training datasets employed for each tool and evaluated on a common blind test set. (b) In all comparisons, MiRduplexSVM shows superior performance, achieving up to a 60% increase in prediction accuracy for mammalian hairpins and can generalize very well on plant hairpins, without any special optimization. (c) The tool has a number of important applications such as the ability to accurately predict the miRNA or the miRNA*, given the opposite strand of a duplex. Its performance on this task is superior to the 2nts overhang rule commonly used in computational studies and similar to that of a comparative genomic approach, without the need for prior knowledge or the complexity of performing multiple alignments. Finally, it is able to evaluate novel, potential miRNAs found either computationally or experimentally. In relation with recent confidence evaluation methods used in miRBase, MiRduplexSVM was successful in identifying high confidence potential miRNAs.

## Introduction

MicroRNAs are small (18–27 nucleotides long), single stranded RNA molecules found in plants, animals, and some viruses. They are most known for controlling protein synthesis either by translation repression or mRNA degradation but they also promote histone modification and DNA methylation of promoter sites, which influence the expression of target genes [1, 2]. Their function is accomplished by binding to the target mRNA through sequence complementarity rules, usually in the 3′untranlated region (UTR) [3]. MicroRNA genes are typically transcribed by RNA polymerase II forming the primary miRNA, often denoted as *pri-miRNA*. Pri-miRNAs are usually several kilobases long and contain local stem-loop structures which are termed *hairpins* [4]. A typical hairpin consists of a stem of base pairs, a terminal loop and two flanking ssRNA segments. In animals the ssRNA segments are detached producing the *pre-miRNA* with a characteristic 3′ overhang of 2nts [4]. This takes place in the nucleus by the Microproccessor complex whose core component is Drosha, an RNAase III type protein [4]. The pre-miRNA is exported in the cytoplasm where is cleaved by an RNAase III type protein, Dicer, producing the *miRNA duplex* with a 3′ overhang of ~2 nts. This cleavage occurs at ~22 nucleotides from the overhang created by the Microprocessor [5]. Finally, one of the two strands of the duplex is selected to exert the function of the miRNA *(mature miRNA)* and the other one is degraded, (miRNA*). There are several examples however where both strands of the duplex correspond to a mature miRNA but only one becomes functional each time [3].

Given the importance of miRNAs in gene regulation, several high throughput experimental approaches such as tiling arrays and deep sequencing are being used in combination with computational methods for the identification of novel miRNA genes and mature miRNAs [6–8]. These methods are particularly useful as they can provide a very sophisticated and accurate expression map for possible miRNA genes in the genome. However, they are also limited by their high cost as well as their tissue and condition specificity. Furthermore, identification of a small RNA sequence in deep sequencing data is not sufficient to categorize this molecule as a true, functional miRNA. As recently discussed by miRBase, additional processing is required in order to increase the confidence of a newly discovered potential miRNA, by identifying for example the respective hairpin(s) and the miRNA duplex, among other features [9]. Such a limitation can be overcome by computational tools, which handle deep sequencing data and/or assess miRNA duplexes or mature molecules, thus facilitating the rapid and precise detection of novel miRNAs. Several such computational approaches have been developed to complement experimental ones including [10–13], among others. However the results are amenable to improvement as performance accuracy with respect to the identification of the exact mature miRNA molecule remains noticeably low and similar to a trivial classifier [14].

In this paper, we focus on the identification of the miRNA duplex given its hairpin sequence. We use this approach because (a) production of the duplex is an essential intermediate stage in miRNA biogenesis and (b) given the duplex it is feasible to experimentally identify the strand(s) producing the mature miRNA molecule. We present a methodology that uses an appropriate representation of biological features combined with extensive optimization and training of SVM classifiers in order to generate predictive models of the miRNA:miRNA* duplex position on a hairpin sequence. It should be noted that a preliminary version of this methodology is described in [15]. Here we extensively optimize SVM’s hyper-parameters and we show that our method outperforms four existing tools, namely MaturePred [11], MiRPara [13], MatureBayes [12] and MiRDup [10] as well as a Simple Geometric Locator [14], a trivial method employing the position as the only predictor used for a baseline comparison. Several factors contribute to the success of the methodology: the definition of the problem (predicting the whole duplex vs. a single strand or end), the representation of the sequence with a fixed-length vector using zero padding in the middle, the inclusion of the duplex flanking sequences, the production of positive and negative training examples based on biological constraints and not the simple 2nt overhang rule, the optimization of the SVM hyper-parameters while avoiding overfitting, and the use of two cost hyper-parameters to address the problem of positive and negative training examples’ imbalance. Moreover, we show that our methodology has a number of important applications besides duplex identification. For example it can be used to accurately predict the miRNA* given a known miRNA molecule, to investigate Drosha processing by introducing mutations in the regions surrounding the duplex [16] and in the evaluation of potential miRNAs found either computationally or via deep sequencing methodologies. The tool is freely available for use as a web-service at http://139.91.171.154/duplexsvm/.

## Methods

The key idea of the proposed methodology is to train and employ a full polynomial SVM model to score each possible duplex position on a hairpin sequence and select the highest scoring one as the final predicted location. The various steps of our methodology are presented in the following paragraphs.

### Candidate Duplex Production

As described in [15], the production of all possible duplexes on a hairpin structure is employed to generate training examples for the SVM during the training phase but also to produce all duplexes to be scored at prediction time; the highest scoring one is the final prediction.

Briefly, the counting of nucleotide positions in a hairpin sequence starts at the 5′ end and continues to the 3′ end. A hairpin is defined as a double-stranded molecule, termed the stem, the two strands of which are connected on one side through a sequence of unmatched nucleotides that form the terminal loop. The strand located before the terminal loop is termed the 5′ arm while the other strand is termed the 3′ arm of the hairpin. Due to their non-perfect complementarity, several small loops and bulges can be found in the two arms.

**Fig 1:**
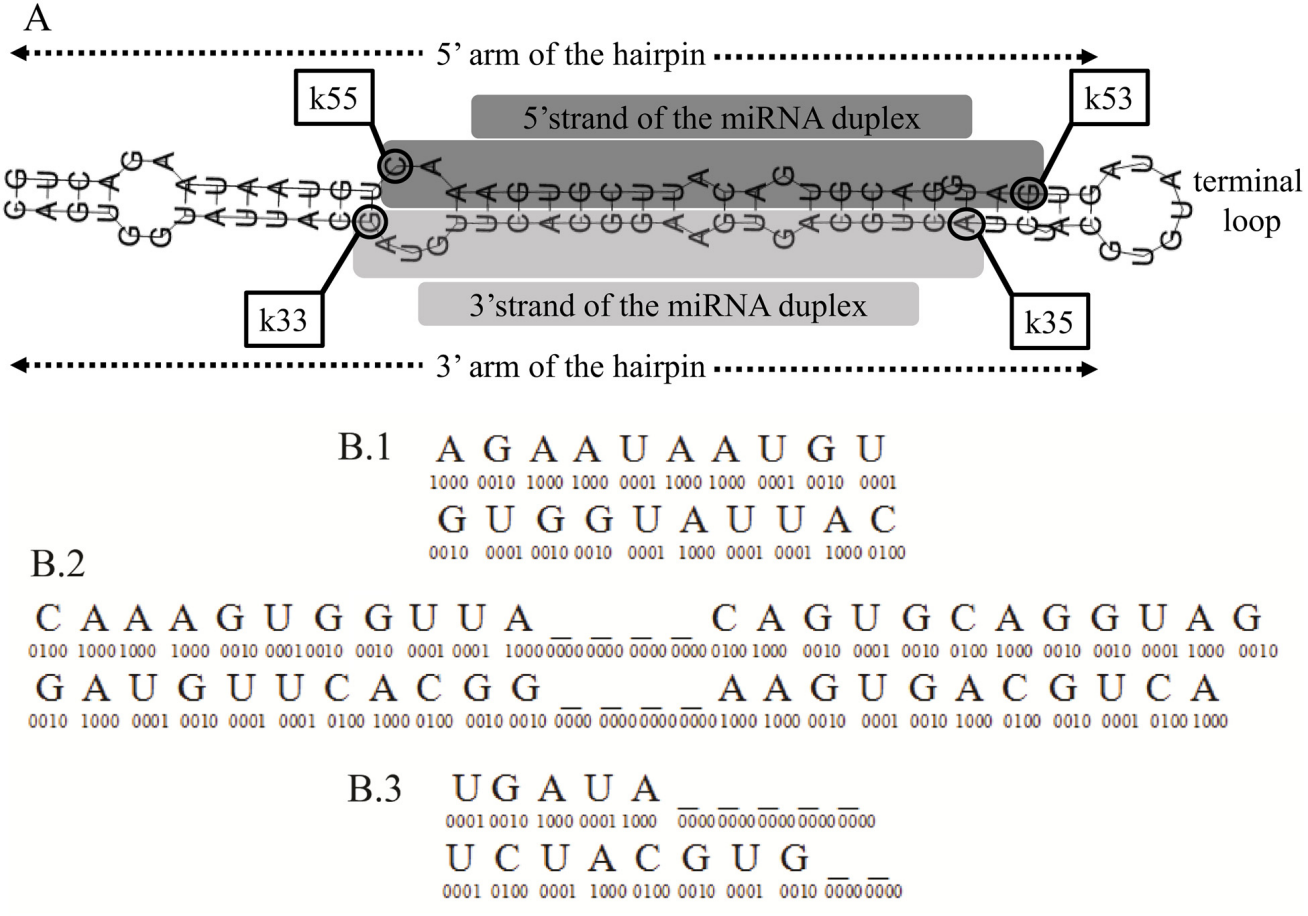
hsa-mir-17 example. A. Anatomy of the hsa-mir-17 hairpin, showing the duplex (grey)and its four ends (k55, k53, k33, k35). B. Vector representation of the true miRNA duplex of the hsa-mir-17 with 10 nucleotides flanking region. B.1. Flanking region of 5′ strand 5′ end (top), and 3′ strand 3′ end (bottom), no zero padding needed. B.2. Duplex sequence, 5′ strand (top), 3′ strand (bottom). Zero padding occurs in the middle to reach the maximum (27nts top, 26nts bottom) length observed in the training set. B.3 Flanking region of 5′ strand 3′ end (top), and 3′ strand 5′ end. Zero padding is performed at the end as flanking region extends beyond the loop tip. Adopted from [15] with modifications.

The miRNA duplex consists of two substrings, the *5′ strand* and the *3′ strand*, which originate from the 5′ and the 3′ arm, respectively. We define a duplex by the positions of its four ends, *k55*, *k53*, *k35*, *k33*, on the generating hairpin sequence. *k55* corresponds to the 5′ end of the 5′ strand, *k53* corresponds to the 3′ end of the 5′ strand, *k35* corresponds to the 5′ end of the 3′ strand and *k33* corresponds to the 3′end of the 3′ strand (see Fig 1A). In addition given that the counting of nucleotide positions starts from the 5′ end and continues to the 3′ end, *k55 < k53 < k35 < k33* holds.

Not all possible substrings on the two strands define a possible duplex. Several constraints that are obeyed by Nature (as far as we know) need to be satisfied: (1) Two strands that share no matching bases do not form a possible miRNA:miRNA* duplex. (2) The length of each duplex strand should lie within a certain range, which can be deduced from known miRNAs. (3) The duplex overhangs (see S1 Text for details) should also lie within specific ranges, which can be calculated using the training examples. (4) *k55 < k53 < k35 < k33* and (5) *k55*, *k53* and *k35*, *k33* need to be before and after the tip of the loop (see S1 Text for tip identification), respectively.

To assemble and detail the whole procedure together, on a given training set, we first predict the secondary structure of each hairpin using the RNAfold program [17] with the default parameters (-p-d0-noLP-noPS), Vienna RNA Package, version 1.8.5. Subsequently, we calculate the statistical distributions of the overhangs’ and matures’ lengths and remove the overhangs’ outlier values (values that are above or below three times the standard deviation from the mean value). This is necessary to reduce the number of candidate duplexes, stemming from values that are too extreme and uncommon. Finally, we produce all duplex sequences that correspond to each combination of values *k55*, *k53*, *k35*, *k33* that obey the constraints defined above. Contrary to earlier work [13], the *k53* end can be positioned as far as the loop tip, allowing the identification of mature miRNAs that extent into the terminal loop.

This methodology results in the generation of ~10,000 candidate duplexes per hairpin, only one of which is the true duplex. During training, true duplexes are labeled positive and the rest form the negative examples. During testing, the true duplex is occasionally not produced due to the restrictions on the possible ranges of the overhangs described above. In the experiments reported here, loss of true duplexes due to this filtering never exceeded 4%.

### Duplex Vector Representation

Extensive experimentation was first performed in order to find the minimum set of features like sequence, structure or thermodynamics needed to obtain maximum accuracy (see Figures F2 and F3 in S1 Text for a comparison of models using various features). Use of sequence information alone was found to be sufficient. Thus, similarly to a preliminary version of the algorithm [15], miRNA:miRNA* duplexes used as input to the SVM are represented by a fixed-length numerical vector that contains only nucleotide sequence information. Briefly, nucleotide bases A, T, G and U are represented by four binary variables as 1000, 0100, 0010 and 0001, respectively. This specific encoding is known as distributed encoding in Machine Learning [18] and was selected for theoretical reasons in an effort to facilitate detection of patterns by the classifier (for details see the Feature Encoding section in S1 Text). Furthermore, since strand sequences are of variable size, the fixed-length numerical vector representation becomes problematic. In order to overcome this difficulty the maximum possible strand length was identified and we padded with zeros at the end for the missing nucleotides. Zero padding was performed in the middle of a sequence, so that the first and the last variables always represent the first and the last nucleotide, respectively. We used this approach because it was previously shown that the end structure and sequence is the primary determinant of Dicer specificity and efficiency [5]. In signal processing, zero padding is common and even though there may be more efficient ways to treat missing information, it does not affect the estimation of model performance or invalidates any results.

As the flanking regions around Drosha and Dicer cut sites are critical for the identification of these sites [19], [12], we include the flanking regions at both ends of each duplex strand in the representation of a candidate duplex. Again, zero padding is employed in the cases where the flanking regions extend beyond the arm boundaries. Specifically we pad with zeros at the beginning or at the end for the 5′ end flanking region and for the 3′ end flanking region, respectively. An example of the miRNA:miRNA* duplex representation for the hsa-mir-17 hairpin is shown in Fig 1B.

### Training and Testing Procedures

**Fig 2:**
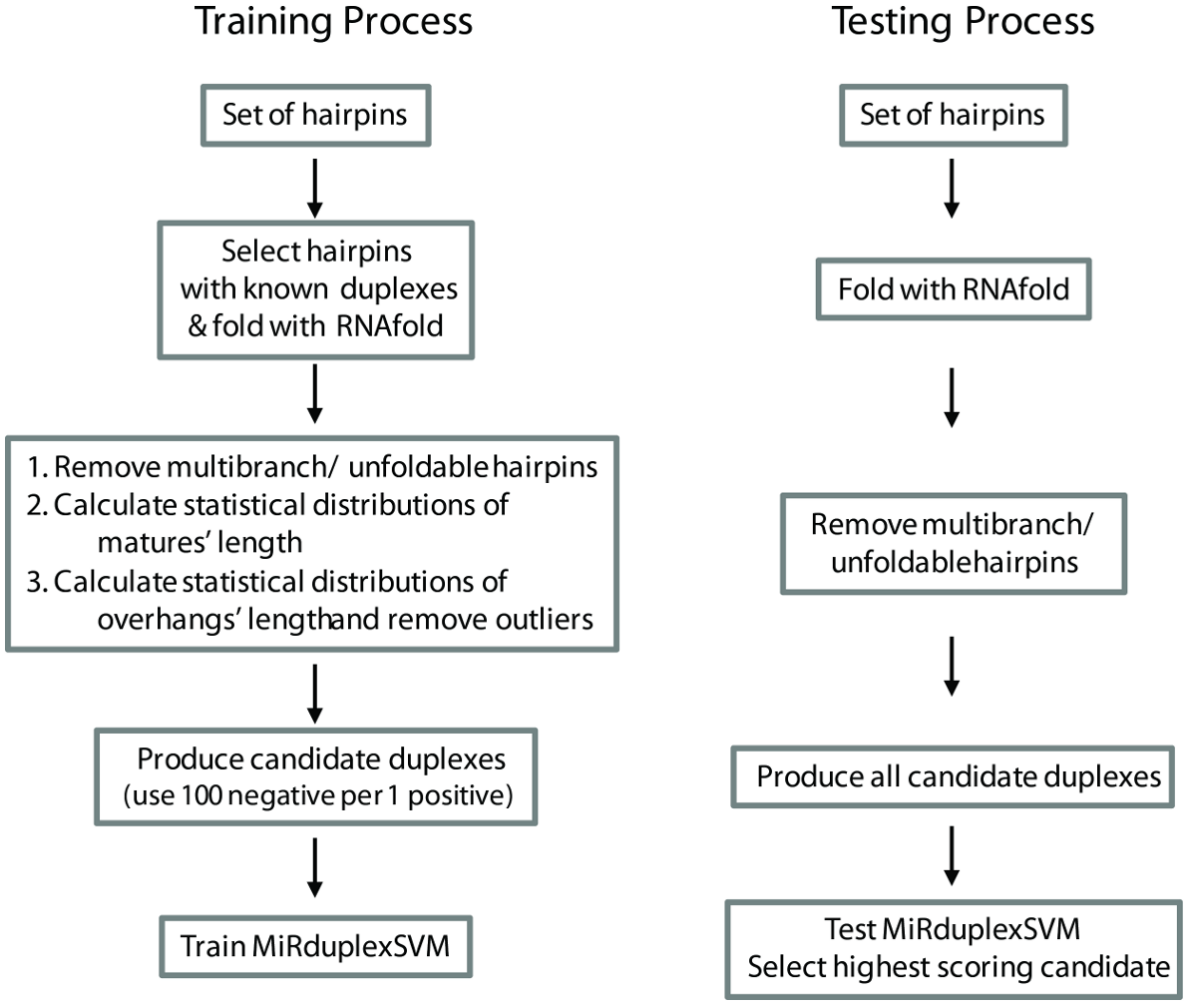
Flowcharts of the training and testing procedures.

The training and testing procedures are depicted in Fig 2. Briefly, given a set of training hairpins, the process consists of the following steps: (a) all entries for which (i) the miRNA:miRNA* duplex is not known or (ii) the RNAfold does not produce a hairpin (unfoldable) or (iii) the produced structure is a hairpin with multiple branches (multi-branch), are filtered out. (b) For each hairpin, all possible duplexes (~10,000 per hairpin) are generated and divided into the single Positive (experimentally verified duplex) and Negative (the rest) examples. To reduce training time, only 100 randomly selected negative duplexes per positive sample are used for training. (c) Selected positive and negative duplexes are used to train an SVM classifier with a full polynomial Kernel *K(x*_*i*_, *x*_*j*_ *) = (x*_*i*_ *• x*_*j*_ *+1)* ^*d*^, where • represents the inner product of the vectors and *d* is the degree of the polynomial. The distribution of the two classes is quite unbalanced (1:100) and special handing is required. The standard (1-norm soft-margin) SVM objective function to minimize is ${\left|\left|\text{w}\right|\right|}_{2}^{2}+\text{c}\underset{\text{i}}{\sum }\left|\xi \_\text{i}\right|$, where ξ_i_ = max(0,1-y_i_(wx_i_+b)) is called the Hinge loss of the *x*_*i*_ input vector, thus giving equal cost weight *c* to the loss of any training vector regardless of its class. We use instead a small modification typical for imbalanced classes (also available in the implementation of LIBSVM that we’ve employed) and minimize ${\left|\left|\text{w}\right|\right|}_{2}^{2}+{\text{c}}_{1}\underset{\text{i}}{\sum }\left|\xi_{\text{i}}^{+}\right|+{\text{c}}_{2}\underset{\text{i}}{\sum }\left|\xi_{\text{i}}^{-}\right|\;$ where $\xi_{\text{i}}^{+},\xi_{\text{i}}^{-}$ are the losses corresponding to the positive and negative examples respectively and *c*_*1*_ *and* c_2_ the penalty weights for the loss of each class respectively. These are computed as *c*_*1*_ = c*N/p and *c*_*2*_ = c*N/n, where *p* and *n* are the number of positives and negative examples respectively, and *N = p+n* the total sample size and *c* is the cost hyper-parameter. To utilize these penalties in LIBSVM we set the parameters *-wi weight* to *w*_*1*_ *= c*_*1*_ for the positive class and *w*_*2*_ *= c*_*2*_ for the negative class. The reasoning behind the penalty weights is that they are proportional to the overall cost *c*, and inversely proportional to the prevalence of each class. Thus, the Hinge loss for examples of the rare class (positives) is higher than the loss for examples of the abundant class. The SVM software used is the MATLAB interface for LIBSVM (version 3.11) [20].

Testing follows a similar procedure: entries for which the RNAfold output is not a hairpin or consists of multiple stems are first filtered out. Then, per-hairpin, all candidate duplexes are generated (note that the ranges of the overhang’s and mature’s length are always deduced from the training set alone). The duplex with the maximum SVM score is selected as the algorithm’s final prediction. A general description of training and testing processes can also be found at [15].

**Prediction error** is assessed using two metrics (see S1 Text): (a) The ACSAE (All Corners Sum Absolute Error) is the sum of absolute errors in number of nucleotides from true position between the actual and the predicted duplex end, taken over all four ends of the duplex. (b) The EAE (End Absolute Error) focuses on a specific end of the duplex; it is the absolute value of the predicted minus the true position (in nucleotides) in a specific duplex end.

To measure **prediction accuracy**, we define as “correct” a prediction with error less or equal to a number *x*. Then, the prediction accuracy for an error bound of at most *x*, denoted as Accu(*x*), is the percentage of correct predictions in the test set. For example, if a model identifies correctly the position of 50% of duplexes with ACSAE ≤ 4, it has accuracy at 4nt of 50%: Accu(4) = 0.5.

**Statistical significance** of the results is assessed by assuming the null hypothesis that two methods have the same accuracy for a given error bound and applying Fisher’s exact test.

### Optimization of SVM Hyperparameters

The method has three hyper-parameters to optimize: the cost *c*, the degree of the kernel *d* of the SVM, and the length of the flanking region *l* (number of nucleotides before and after the duplex) of the vector representation. We note that proper selection of these hyper-parameters was critical to achieving high performance. They were optimized once using 5-fold cross validation on a randomly-selected subset of version 17.0 of miRBase, consisting of 70% (658 in number) human/mouse hairpins with known duplexes. The values tested were: *d* = 1, 2, 3, *1/c* = 100, 10, 1, 0.1, 0.01, 0.001 and *l* = 0, 3, 6, 9, 10…15 nts. The performance during cross-validation was measured in terms of predicting the exact location of the duplex by calculating the sum of the absolute error taken over all four ends of the duplex (ACSAE). The best performing combination of parameters found was *d* = 3, *1/c* = 0.01 and *l* = 10nts. This combination of parameters was employed in all MiRduplexSVM models reported here. *To ensure unbiased estimations of performance*, *the 658 hairpins used for hyper-parameter optimization were excluded from all test sets used in subsequent evaluations*.

## Results

### Comparison with a Simple Geometric Locator

**Fig 3:**
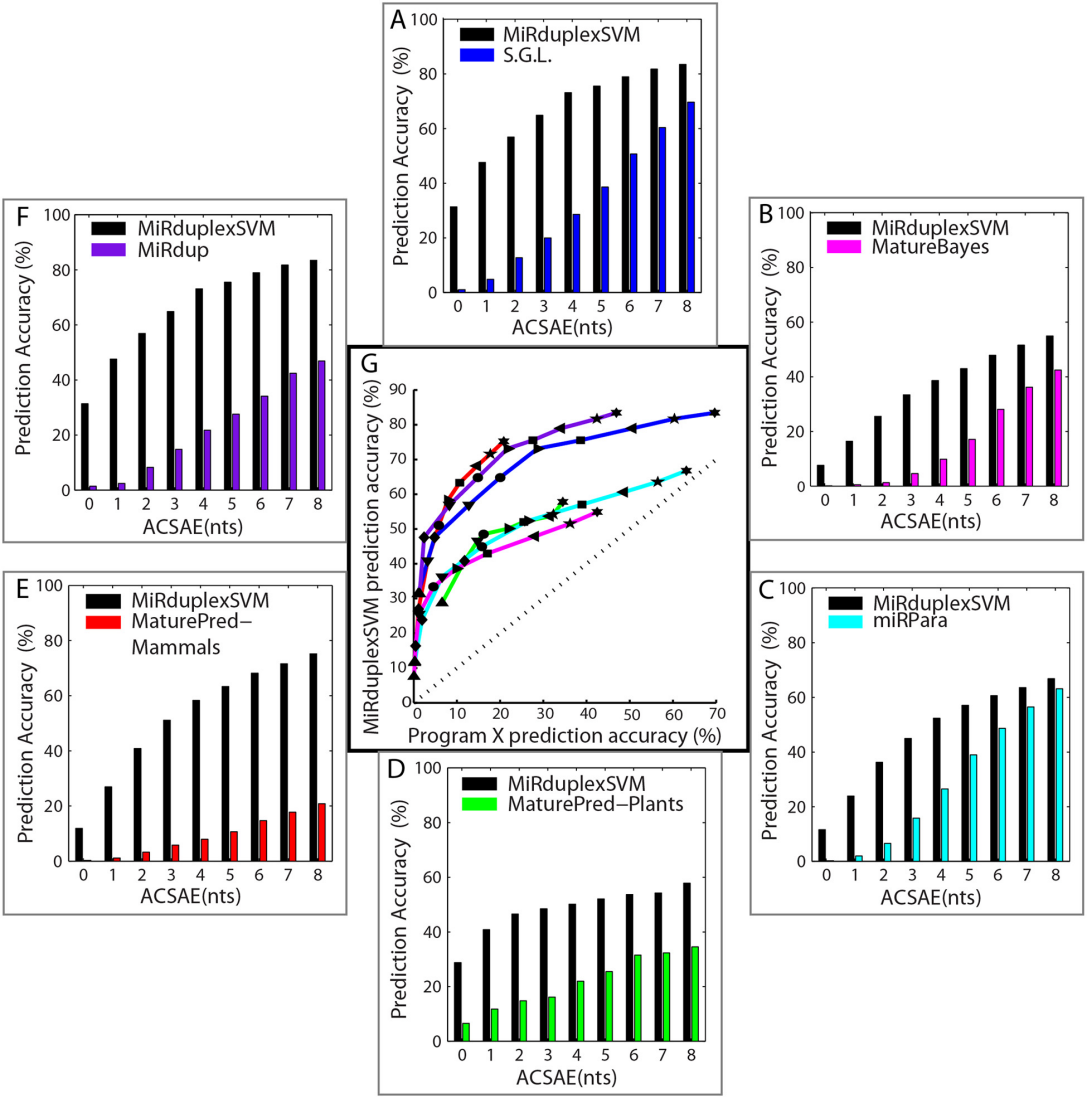
Prediction accuracy of MiRduplexSVM and six other methods on duplex identification. Panels A-F show the prediction accuracy (y-axis) of MiRduplexSVM (in black) and a second compared tool (in colour) as a function of the All Corners Sum Absolute Error (ACSAE, x axis) for errors of 0-8nts. The performance of the Simple Geometric Locator (S.G.L.), MatureBayes, miRPara, MaturePred-Plants, MaturePred-Mammals and MiRdup is shown in A—blue bars, B—pink bars, C—cyan bars, D—green bars, E-red bars and F-purple bars, respectively. Panel G shows the prediction accuracy of MiRduplexSVM (y axis) against the prediction accuracy of each compared tool (x axis). The colour code is the same as in A-F. Symbols (upward triangle, diamond, downward triangle, circle, right pointed triangle, square, left pointed triangle, pentagram star and hexagram star) correspond to errors less than or equal to 0, 1, 2, 3, 4, 5, 6, 7, 8 nucleotides, respectively. All points above the diagonal in G are statistically significant at level 0.05.

**Fig 4:**
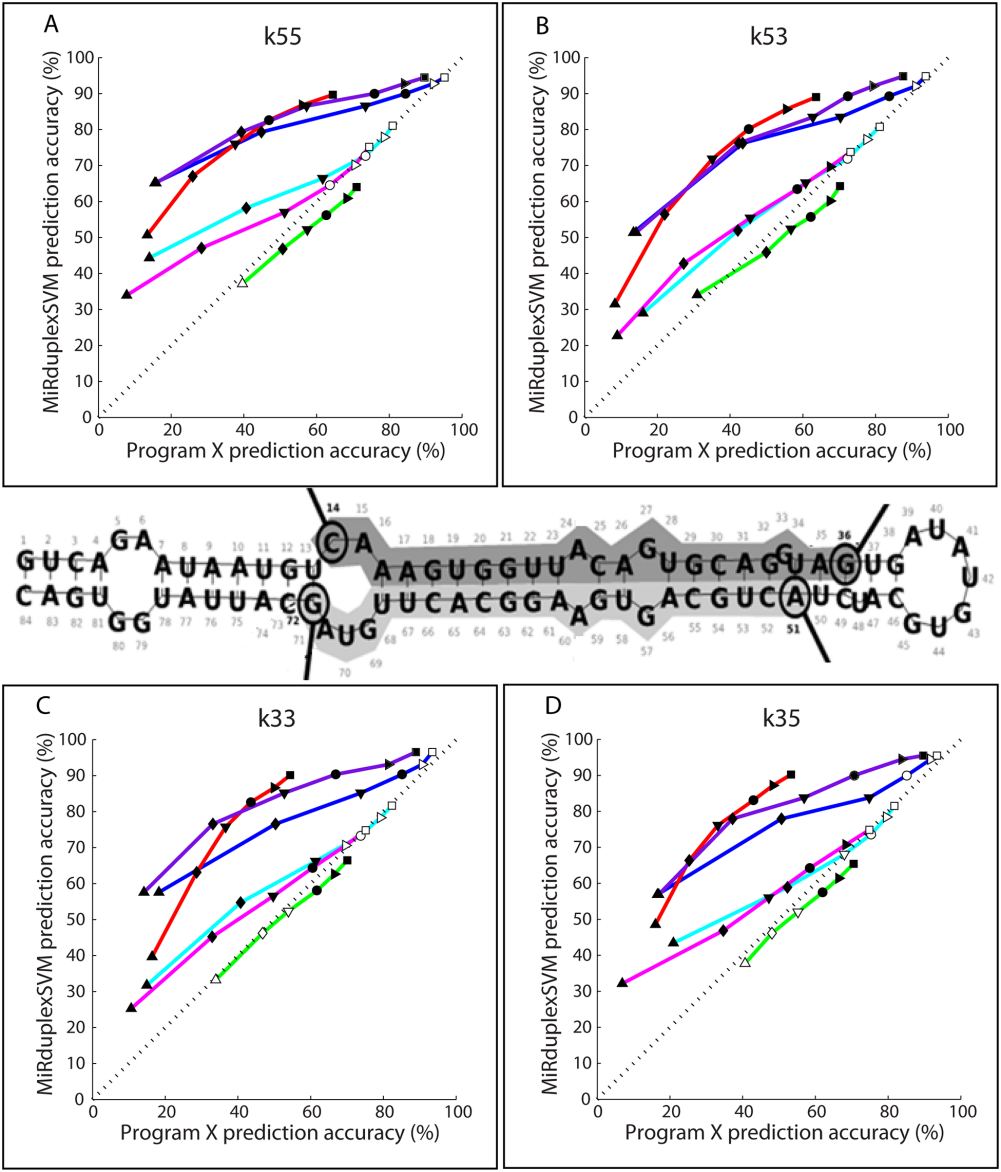
Prediction accuracy of MiRduplexSVM versus six other methods on corner identification. Performance accuracies are estimated using the EAE for errors of 0-5nts. In each panel, the y axis shows the prediction accuracy of MiRduplexSVM (in %) and the x axis shows the prediction accuracy of other methods (in %). The colour and symbol scheme is the same as in Fig 3G. Statistically significant results are indicated with filled symbols.

The performance of MiRduplexSVM was first compared to that of the Simple Geometric Locator (S.G.L.), the construction of which is detailed in [14]. Briefly, in the S.G.L., the location of each of the four ends of known duplexes is found by calculating its distance from the tip of the terminal loop. This is done for all hairpins in the training set and the average distances (rounded to the closest integer) are then used to generate the predictions of the S.G.L. for any new hairpin in the test set. In the comparison reported here, both methods were trained on the dataset used to optimize the hyper-parameters of MiRduplexSVM and tested on the remaining 30% of hairpins with known duplexes (290 hairpins) in version 17.0 of miRBase. Fig 3A shows the prediction accuracy of each tool as a function of the ACSAE while Fig 3G (blue line) and Fig 4 (blue lines) show the prediction accuracy of MiRduplexSVM against that of the S.G.L. estimated using the ACSAE (0-8nts, Fig 3G) or the EAE (0–5nts, Fig 4), respectively. In all cases MiRduplexSVM greatly outperforms the S.G.L., especially for small error values. The observed difference in performance is statistically significant for ACSAE of 0–15 nucleotides (see Table T1 line 6, in S1 Text; p = 0.05), and for EAE of 0–3 nucleotides (see Table T2, lines 21–24 in S1 Text; p = 0.05), beyond which both methods behave similarly. Having shown that the distance from the tip loop, while a very simple approach, is not sufficient to identify miRNA duplexes, we next compare MiRduplexSVM with existing miRNA mature prediction tools.

### Comparison with other State of the Art Tools

In the following paragraphs we compare MiRduplexSVM with four state-of-the-art mature miRNA prediction tools, namely MatureBayes [12], MiRPara [13], MaturePred [11] and MiRdup [10]. To ensure fairness, in each comparison MiRduplexSVM is trained with the original training set of the compared tool and evaluated on a common hold-out test set. The procedure for building the various test sets is depicted in Figure F4 in S1 Text and the details on how training/testing was performed for each tool can be found in S1 Text. Performance accuracies on (a) duplex identification, using the ACSAE, and (b) independent corner identification, using the EAE were estimated only on hairpins that are predicted to contain a mature miRNA by both of the compared tools. Comparisons shown in Figs 3 and 4 are performed by finding the prediction accuracy of each tool for an ACSAE of 0-8nts and an EAE of 0-5nts and (a) plotting these accuracies as a function of the ACSAE metric (Fig 3A–3F) or (b) plotting these accuracies against each other (Fig 3G and Fig 4). Specifically, on Fig 3G the prediction accuracy, measured as the ACSAE (in %), of MiRduplexSVM (y axis) at error points 0-8nts is plotted against the respective accuracy of each compared tool (x-axis). For example, the first point (triangle) on each line represents the pair of accuracies (Accu_i_(0), Accu_MiRduplexSVM_(0)), the second point (rhombus), the pair (Accu_i_(1), Accu_MiRduplexSVM_(1)) and so on, where *i* is the compared tool. The points that correspond to comparisons against a given tool *i* are connected with a line. Thus, if a line is right on the diagonal, then the two methods achieve the same accuracy for the same error tolerance. If it is above the diagonal, then MiRduplexSVM achieves the same accuracy for smaller error levels than the method compared against. The same applies to Fig 4, with the only difference that the accuracy is measured using the EAE instead of the ACSAE. The results of each tool comparison are summarized below.

*MatureBayes* was the first tool specifically developed to address the problem of mature miRNA identification. Comparison to MiRduplexSVM is shown in Fig 3B and 3G (pink line) for duplex prediction and Fig 4 (pink lines) for independent corner prediction. As evident from the figures, MiRduplexSVM significantly outperforms MatureBayes (pink lines are above the diagonal) in both duplex (up to 12 nts, see Table T1, row 1 in S1 Text) and independent corner (up to 4nts, see Table T2, rows 1–4 in S1 Text) prediction.

*MiRPara* is an SVM-based tool for mature miRNA prediction [13]. Comparison to MiRduplexSVM is shown in Fig 3C and 3G (cyan line) for duplex prediction and Fig 4 (cyan lines) for independent corner prediction. It should be noted that MiRPara gave a prediction for only 3,774 out of the 5,000 hairpins used for testing. Prediction accuracy for both models was calculated on these 3,774 hairpins, which biases the comparison in favour of MiRPara. As evident from the figures, MiRduplexSVM significantly outperforms MiRPara (cyan lines are above the diagonal) in both duplex (up to 8nts, see Table T1, row 2 in S1 Text) and independent corner (up to 2nts, see Table T2, rows 5–8 in S1 Text) prediction. Note, that the S.G.L. also has a good performance for errors beyond 3-4nts (see Table T2 in S1 Text) indicating that even the simplest method can find the true mature when the tolerance for errors is more than a couple of nucleotides per corner.

*MaturePred* [11] calculates the region where the mature miRNA molecule is more likely to be found in each strand of a hairpin, using an SVM based approach. It consists of two models, one specialized in plants, hereby termed MaturePred_Plants and one specialized in mammals, hereby named MaturePred_Mammals. We compare MiRduplexSVM with each model separately. Comparison to MiRduplexSVM is shown in Fig 3D (Plants), 3E (Mammals) and 3G (Plants: green line, Mammals: red line) for duplex prediction and Fig 4 (Plants: green lines, Mammals: red lines) for independent corner prediction. As evident from Fig 3D, 3E and 3G (lines above diagonal), MiRduplexSVM significantly outperforms MaturePred on duplex prediction for both plant and mammalian hairpins (for all errors tested, see Table T1, rows 3 & 4 in S1 Text). For independent corner prediction, MiRduplexSVM outperforms MaturePred in a statistically significant manner only for mammalian hairpins (red lines above diagonal), while for plant hairpins both tools achieve similar performances (Fig 4, green lines on the diagonal and Table T2, rows 9–16 in S1 Text).

*MiRdup* [10] is the latest tool that tackles the problem of mature miRNA identification. It does so by finding the most likely miRNA location within a given pre-miRNA. Comparison to MiRduplexSVM is shown in Fig 3F and 3G (purple line) for duplex prediction and Fig 4 (purple lines) for independent corner prediction. As evident from the figures, MiRduplexSVM significantly outperforms MiRdup (purple lines are above the diagonal) in both duplex (up to 20nts, see Table T1, row 5 in S1 Text) and independent corner (up to 6nts, see Table T2, rows 17–20 in S1 Text) prediction.

In sum, on the task of duplex prediction, MiRduplexSVM outperforms all other tools it has been compared to for an error tolerance of at least 8nts and the increase in performance accuracy ranges from ~10% to 60% (Fig 3G and Table T1 in S1 Text). With respect to individual end comparisons (Fig 4 and Table T2 in S1 Text), MiRduplexSVM is again found to outperform all methods, particularly for small EAEs (0-4nts). The only exception is MaturePred-Plants which achieves a similar performance. The latter maybe due to the parameter optimization of MiRduplexSVM which was done using mammalian hairpins and/or the small number of plant hairpins used to train MiRduplexSVM (198) compared to MaturePred_Plants (1,323).

### Final model

Having established the superiority of our algorithm compared to existing tools, we generated a final model using all hairpins with known duplexes (5,248) available in miRBase 19.0. The model was evaluated on 5,000 randomly selected hairpins from miRBase 19.0 having the same species ratio as the remaining 15,500. Mature sequences in these 5,000 hairpins were equally distributed in both strands. The accuracy of MiRduplexSVM was evaluated using the EAE for each strand independently, since the ACSAE cannot be computed without knowledge of the true duplex. It was found to reach 55%, 39%, 54% and 43% correct prediction at 0 nucleotides deviation for k55, k53, k35 and k33, respectively (see Table 1). This final model achieves higher performance than the one seen in the comparisons with other tools, presumably because it is trained with a much larger training set. The model is available for download at http://139.91.171.154/duplexsvm/.

**Table 1:** End Absolute Error (EAE) in nts.

**Prediction Accuracy(%)**		**≤ 0**	**≤ 1**	**≤ 2**	**≤ 3**	**≤ 4**	**≤ 5**
	k55	55.46	64.66	71.33	75.62	79.44	82.25
	k53	39.4	59.04	70.88	76.14	80.24	82.97
	k35	54	64.85	71.38	76.42	80.1	82.87
	k33	43.35	61.53	70.18	75.58	80.1	82.87

Final MiRduplexSVM model predictions for EAE up to 5nts. The absolute error for each one of the four ends of the duplex is calculated independently.

### Practical applications

We next assessed the applicability of our method on two important problems: (a) the accurate identification of the opposite strand of a known miRNA, (b) the identification of high confidence potential miRNAs identified via deep sequencing or other methodologies.

#### Missing Duplexes Identification

We first tested our methodology on the problem of identifying the mature molecule that lies on the opposite strand of a known miRNA. This is an important problem as both of these molecules are frequently functional, albeit under different conditions, and thus experimental techniques are unlikely to detect them both in a single experiment. To the best of our knowledge, this is the first attempt to find opposite strand miRNAs using a machine learning approach.

Towards this goal, we set the known miRNA of each hairpin as the ground truth for that strand and produce all candidate duplexes generated by sliding along the opposite strand. The final prediction is the highest scoring candidate. MiRduplexSVM is compared to a simple classifier, termed “Overhangs Ruler”, which uses the statistical distributions of overhang lengths in the training set to identify the most frequently occurring values for 3′ and 5′ strands. In the majority of the cases, these values are equal to 2nts, a number that is commonly used in computational studies to find the miRNA* [11], [12]. For a new test hairpin, the missing strand of the duplex is estimated by assigning the overhang lengths to the known miRNA ends.

Both algorithms were trained on a dataset of 3,248 hairpins (containing a known duplex) and evaluated on a set of 2,000 hairpins (with known duplexes) using the EAE metric. Prediction accuracies were measured for each strand independently and the results are listed in Table 2. MiRduplexSVM was found to outperform the Overhangs Ruler on identifying the start position of the miRNA* (Table 2, rows 1 and 3), while both algorithms achieve the same performance on predicting the end position (Table 2, rows 2 and 4). This finding is probably due to the implementation of a more realistic rule for overhang estimation by MiRduplexSVM, whereby the size of the overhang is allowed to vary within the experimentally reported range (see Figure F1 in S1 Text for overhang distribution).

**Table 2:** End Absolute Error (EAE) in nts.

**Prediction Accuracy of MiRduplexSVM / Overhangs Ruler**		**≤ 0**	**≤ 1**	**≤ 2**
	k55	70 / 56 ***	85/84 ns	91/92 ns
	k53	53 / 53 ns	79/81 ns	90/91 ns
	k35	67 / 53 ***	85/81 ***	91/91 ns
	k33	58/56 ns	82/84 ns	89/92 ns

Missing duplexes prediction results for MiRduplexSVM and the Overhangs Ruler. MiRduplexSVM outperforms Overhangs ruler in the identification of the start position of the mature miRNA that lie on the 5′ or the 3′ strand, but achieves the same accuracy on the prediction of their end positions. Statistical significance was assessed using the Fisher exact test.

*** corresponds to p-value ≤ 0.001

** to p-value ≤ 0.01

* to p-value ≤ 0.05 and ns to non significant.

Our predictions were also contrasted to the results of a comparative genomics approach, a method frequently employed to find conserved miRNAs[21, 22]. Opposite strand molecules were identified by searching for orthologs in other species, utilizing the gene name of each miRNA. Orthologs with known duplexes were used to predict opposite strand miRNAs as long as (a) the known miRNAs were exactly the same across species and (b) the sequence of the opposite strand molecule was part of the hairpin under investigation. It is important to mention that if more than one orthologs met these requirements, several predictions were produced per hairpin. In this case, only the prediction with minimum EAE was used for comparison and thus the results provide an upper bound of the performance using orthologs based on best-case analysis. This process resulted in the identification of opposite strand miRNAs for 30 genes, while we note that the MiRduplexSVM is capable of providing predictions every time. When compared to the MiRduplexSVM predictions for the same hairpins using the EAE metric, both methods gave the same predictions within a window of 2nts deviation (Table 3).

**Table 3:** End Absolute Error (EAE) in nts.

**Prediction Accuracy (%) of MiRduplexSVM with respect to the comparative genomics results**		**≤ 0**	**≤ 1**	**≤ 2**
	k55	81.82	95.45	100
	K53	63.64	95.45	100
	k35	85.71	100	100
	k33	100	100	100

MiRduplexSVM versus comparative genomics on missing duplexes prediction. The table shows the prediction accuracy per corner of MiRduplexSVM when the results of a comparative genomics approach are set as the ground truth. When considering an error tolerance of up to 2nts, MiRduplexSVM gives exactly the same predictions as a strict comparative genomics algorithm.

Finally, we used MiRduplexSVM to predict all missing duplexes of human and mouse hairpins (1,240 mature miRNAs, see S2 Text).

#### Identification of high confidence potential miRNAs

**Fig 5:**
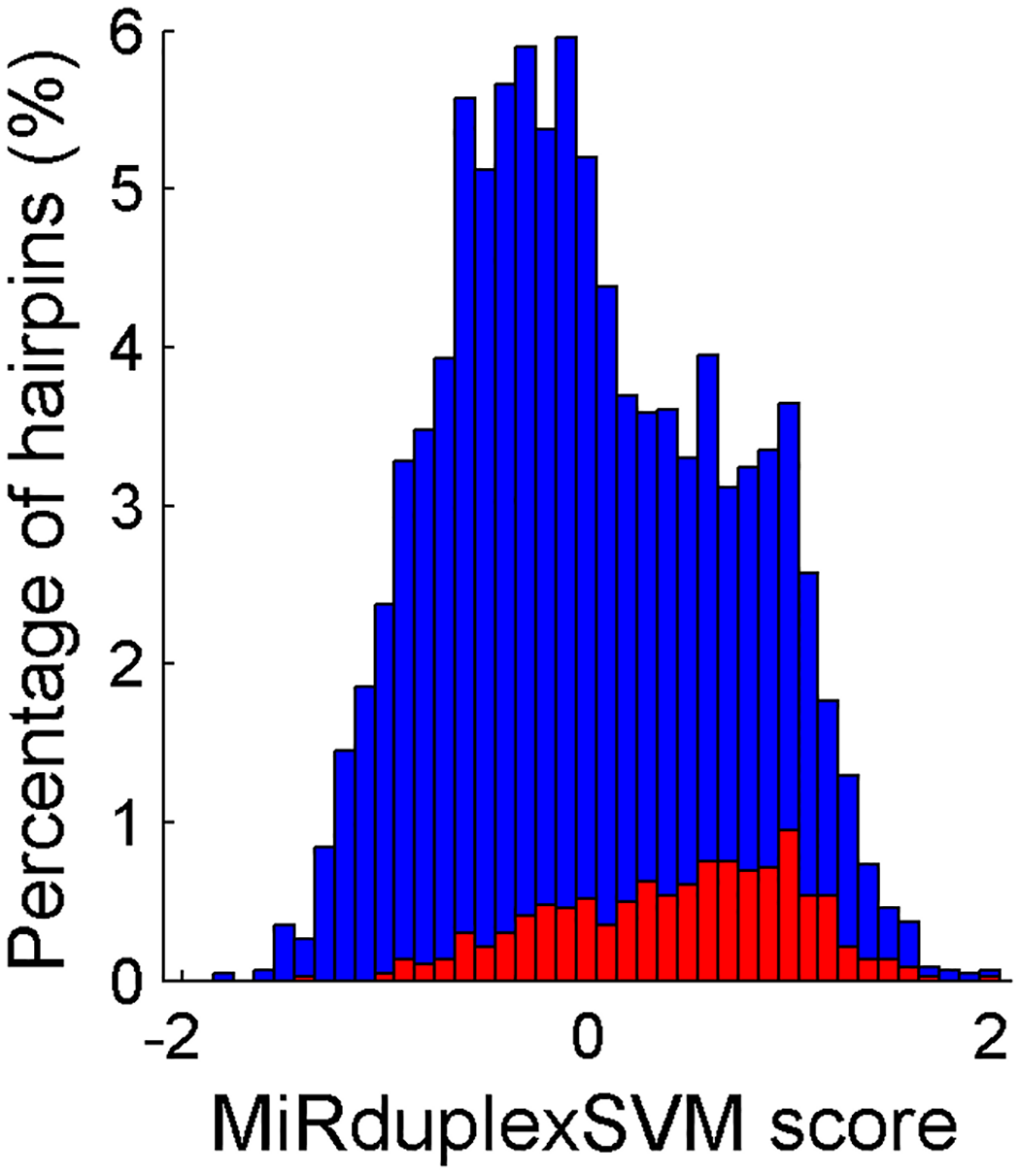
Identification of high confidence miRNAs. As shown in the figure MiRduplexSVM assigned a higher score to 554 high confidence miRNAs (blue bars, median = 0.53 and mean = 0.44) than to 554 randomly selected miRNAS (red bars, median = -0.24 and mean = -0.14) with the observed differences being statistically significant (ranksum: p = 8.3084e-47 and t-test: p = 3.9577e-50). The x axis shows MiRduplexSVM’s scores and the y axis shows the percentage of hairpins assigned with the respective scores.

The issue of identifying high confidence miRNAs in its large number of entries was recently raised by miRBase, the largest miRNA database [9]. To address this important problem, miRBase defined a set of five criteria that need to be met in order to characterize an entry as a high confidence potential miRNA. One of these criteria refers to the duplex conformation and uses a more flexible version of the 2nts overhang rule. We tested the performance of our final model on the problem of identifying high-confidence miRNAs from miRBase as reported in [9]. MiRNAs used in the training of our model were excluded from this analysis. As shown in Fig 5, the MiRduplexSVM's scores assigned to high-confidence miRNAs (554 hairpins) are localized in the right part of the overall MiRduplexSVM scores' statistical distribution, which was estimated by scoring miRNAs (4,000) belonging in the same species as the high-confidence ones. Their localization was highly statistically significant (ranksum test, p = 4.3558e-45). These results suggest that MiRduplexSVM can also be used to identify the most promising, newly discovered miRNA candidates.

## Discussion

In this paper we address the problem of predicting and evaluating the miRNA:miRNA* duplex stemming from a miRNA hairpin as a first step in identifying the mature miRNA(s); the latter is important both for experimentally verifying the miRNA and for computationally predicting target mRNAs. We present the need for stringent assessment of deep sequencing data during miRNA discovery, focusing on the duplex evaluation as a means of this implementation. We employ biological knowledge and constraints in converting the problem to a classification one and train a high-order polynomial SVM model to identify the true duplex among numerous candidates.

### Features of MiRduplexSVM that may underlie its high performance

We show that our methodology generates models that outperform a distance based Simple Geometric Locator and four existing miRNA mature prediction tools, namely MatureBayes, MiRPara, MaturePred and MiRdup. Enhancement in performance is very high (10–60%) and seen on both duplex and miRNA or miRNA* prediction. It should be noted that in the comparisons reported here, MiRduplexSVM was trained on the training set of each compared tool to ensure fairness (tools were not available for retraining). It is likely that this improvement would be even higher if the comparison was made against our final model.

The reasons behind this increase in accuracy achieved by MiRduplexSVM are multiple: first, our tool is trained to recognize miRNA:miRNA* duplexes, as opposed to strand-specific miRNAs, which is the standard approach of existing tools [11, 12, 13]. Duplex formation is an indispensable stage in the biogenesis of all miRNAs, regardless of which strand will end up producing the functional molecule [4]. MiRduplexSVM takes into account this biological process and while it does not learn to distinguish which of the two strands is the functional miRNA (this information is often not available), our results show that learning duplexes is a very successful strategy for identifying strand specific miRNAs. Second, our tool learns to identify both the start and the end positions of the miRNA:miRNA* sequences, while most existing tools predict only the starting nucleotide and use a fixed size length of 22nts to find the end position [11, 12]. To achieve this MiRduplexSVM uses a variable length parameter for each miRNA molecule. As a result, MiRduplexSVM does not only outperform other tools in predicting the start position of strand-specific miRNA molecules, but it also succeeds in specifying their length. Third, MiRduplexSVM does not assume a fixed size (2nt) overhang length like most existing approaches [10, 11, 12, 13]. In contrast, due to the duplex generation, the length of each overhang is defined using a simple, yet important algorithm and explicitly learned by the training examples. This feature is likely to also contribute to the algorithm’s success. In addition to the above, there may be other reasons for increased performance. For example, the Naïve Bayes Classifier employed in MatureBayes [12] makes the assumption that each feature (i.e., nucleotide at a given position) is probabilistically independent of each other feature given the class (i.e., whether a sub-sequence forms a mature miRNA or not). This assumption may not be satisfied in these data. In contrast, SVMs make a different set of assumptions which may be more appropriate for this problem; namely, they assume the statistical distributions of the two classes are separated well by a hyper-plane once they are mapped in kernel space. A priori there is no theoretical way for determining which set of assumptions (and which classifier) is better in a given problem. Fourth, the zero padding, flank regions, and the representation to a fixed-vector size ensure that the start and end locations of all mature molecules are represented in the same way, which may also be a performance factor. Fifth, the use of different cost hyper-parameters in the objective function of the SVM to handle the imbalance of the positive and negative classes is also critical for the specific problem. Our experience with training the models shows that optimization and tuning of the SVM hyper-parameters (cost and polynomial kernel degree) is crucial for achieving good performance.

Finally, an important advantage of our model is its simplicity and cost effectiveness that results from the use of sequence information alone, as opposed to structure and thermodynamics that are often used by other tools [11–13]. However, some structural information is implicitly incorporated in the complete procedure that regards the following: (a) selection of the miRNA hairpin sequence by removing multibranch hairpins and unfoldable hairpins during both training and testing, (b) estimation of the ranges of overhangs and mature miRNA lengths, and (c) computation of candidate miRNA duplexes so they observe the overhang and mature miRNA constraints. Thermodynamic features and information is not explicitly employed by any stages of the method. In fact, the incorporation of structural features in the model did not improve performance, as shown in section “Set of features used” in the S1 Text. There are two explanations why these features do not improve performance. The first explanation is that the predictive information they carry is contained in the sequence features and the classification methods we use are able to capture (learn) this information thus making the structural and thermodynamic features superfluous for prediction. A second explanation is that the latter features do carry additional predictive information, but the classifiers we employed cannot capture it. Thus, they seem superfluous. It is impossible to distinguish between the two scenarios in general, but given the success and accuracy of the models we used, as well as the diligence in optimizing them and employing several flexible SVM kernels able to detect non-linear patterns, there is enough evidence to claim that the structural and thermodynamic features are superfluous given the sequence features.

### MiRduplexSVM can facilitate the miRNA discovery process

MiRduplexSVM can be used to identify the miRNA:miRNA* duplex of a miRNA gene given the hairpin sequence. The hairpin sequence does not need to be precisely defined; it may be generated by one of the numerous computational tools that predict miRNA genes [23–27]. Such an approach is useful when searching for novel miRNAs that may be involved in a particular phenotype. For example, we recently developed and used a miRNA gene finding tool to locate potential new miRNAs residing in cancer associated genomic regions [24]. Similar efforts have been reported in a number of other studies, where new miRNA genes were computationally predicted [26, 28]. In order to verify that a predicted miRNA gene/hairpin produces a functional miRNA however, a number of wet-lab experiments must be performed, requiring significant amount of time, money and effort [29]. MiRduplexSVM can provide reliable predictions about the most likely sequence of the miRNA molecule in these cases, thus guiding experimental efforts and ultimately reducing working hours and costs.

Another case where MiRduplexSVM would be useful is the in-silico study of factors that determine the cleavage sites of drosha and dicer, which define the miRNA:miRNA* duplex [16]. This could be done by performing *in silico* mutagenesis experiments, generating predictions that can then guide the much more demanding wet—lab mutagenesis experiments [30, 31].

### MiRduplexSVM complements deep sequencing methods

MiRduplexSVM can also be applied in the assessment of confidence for newly discovered potential miRNA sequences which are produced in large amounts by experimental methods such as deep sequencing. Many algorithms focus on the evaluation of the miRNA hairpins in order to categorize deep sequencing miRNA candidates as true miRNAs [6]. While this is a valid approach, identification of a hairpin is not sufficient to determine whether a small RNA sequence is indeed a miRNA. Given the cumulating increase in the number of newly discovered potential miRNAs, this issue was also recently raised by miRBase [9]. It is has now become necessary to apply new algorithms that can further process miRBase entries in order to provide confidence scores for each entry. We believe that an integrated miRNA evaluation process should start by applying programs like miRDeep, which utilize deep sequencing data to computationally predict the hairpin sequences that may have produced the respective RNA sequences. A second step should include evaluation of the duplex(es) that can be produced by these hairpins in order to provide additional evidence for the possibility of a given sequence to correspond to a true miRNA. To our knowledge, one other algorithm has tried to address the issue of scoring duplexes for miRNAs evaluation [10], albeit with a much lower performance than the one achieved by MiRduplexSVM or even a trivial classifier[14]. These results suggest that use of MiRduplexSVM for duplex evaluation would greatly facilitate miRNA discovery by taking advantage of the large amounts of experimental data currently available. A third step that would further facilitate the discovery process could include automatic target identification for high confidence miRNAs and search for anti-correlations in expression profiles of miRNAs and their respective targets. Future work could address this challenging problem.

### Conclusions

In conclusion, we present a novel methodology for the computational identification of the mature molecule(s) within novel miRNA hairpins. Our methodology takes into account several aspects of the biogenesis of miRNAs, whereby a duplex is formed before the mature molecule is selected. Our tool is the first that predicts miRNA duplexes and is shown to achieve much higher performance that four existing tools on both duplex and strand-specific miRNA prediction for mammalian hairpins. Moreover, the tool performs equally well on plant hairpins, without any particular customization. Importantly the tool has a number of applications including the identification of opposite strand miRNAs and the evaluation of potential miRNAs detected experimentally through scoring of their computationally identified duplexes. These findings highlight the importance of our method, both as a step forward form the current state of the art in miRNA duplex prediction but also as a useful tool for experimental biologists. The tool is freely available online, via a friendly web interface and can be used either as a web service or a stand-alone application.

## Supporting Information

S1 Text Supporting information. Detailed explanation of MiRduplexSVM’s methodology. S1 Text includes a figure depicting the statistical distribution of the overhangs’ length (Figure F1), a figure depicting the mean prediction accuracies when using different input features (Figure F2), a figure depicting the mean prediction accuracy for the “Sequence” and “Sequence—Entropy” models (Figure F3) and a figure depicting the procedure for building the test sets (Figure F4). S1 Text also includes tables depicting prediction accuracies of the various compared tools for up to 20nts deviation from the truth (Table T1) and up to 8 nts deviation from the truth (Table T2)
(DOCX)

S2 Text List of predicted missing duplexes. MiRduplexSVM was used to predict all missing duplexes of human and mouse hairpins (1,240 mature miRNAs).
(TXT)
