# Supplementary material for: MiRduplexSVM: A High-Performing MiRNA-Duplex Prediction and Evaluation Methodology
Source: PLoS One. 2015 May 11;10(5):e0126151. doi: 10.1371/journal.pone.0126151 (PMC4427487; doi:10.1371/journal.pone.0126151)
Supplement: S1 Text — Detailed explanation of MiRduplexSVM’s methodology. S1 Text includes a figure depicting the statistical distribution of the overhangs’ length (Figure F1), a figure depicting the mean prediction accuracies when using different input features (Figure F2), a figure depicting the mean prediction accuracy for the “Sequence” and “Sequence—Entropy” models (Figure F3) and a figure depicting the procedure for building the test sets (Figure F4). S1 Text also includes tables depicting prediction accuracies of the various compared tools for up to 20nts deviation from the truth (Table T1) and up to 8 nts deviation from the truth (Table T2) (DOCX) [file pone.0126151.s001.docx]

# Supplementary File S1 Text

### Feature Encoding

miRNA:miRNA* duplexes used as input to the SVM are represented by a fixed-length numerical vector that contains only nucleotide sequence information. Briefly, nucleotide bases A, T, G and U are represented by four binary variables as 1000, 0100, 0010 and 0001, respectively. The specific encoding was selected for theoretical reasons in an effort to facilitate detection of patterns by the classifier. This particular encoding is known as distributed encoding in Machine Learning and is suggested in the book Machine Learning, Tom Mitchell, McGraw Hill, 1997, chapter 4 [[18](#_ENREF_18)]. In summary, the selected encoding creates linear separating surfaces for simple OR decision functions. The alternative (binary) encoding introduces non-linearities to simple OR decision functions. For example, suppose that the start of a mature miRNA is determined by whether there is a C or G present at a particular location, i.e., it is a simple OR function. We only consider a single location here for clarity of the example. Thus, the decision surface can be described as f(nucleotide) = sign(C + G – 0.5), where C is 1 if the nucleotide is 1 and G is 1 if the nucleotide is 1. With the distributed representation G is encoded as the feature vector **〈**V1, V2, V3, V4**〉** = **〈**0, 1, 0, 0**〉** and C is encoded as **〈**V1, V2, V3, V4**〉** = **〈**0, 0, 1, 0**〉**. Thus, a function that correctly classifies correct a strand is f(V1, V2, V3, V4) = sign( V2 + V3 – 0.5 ). In other words, the classifier linearly separates the input vectors since V2 + V3 – 0.5 is a linear (technically speaking, an affine) function. In the second encoding, G is represented by the pattern or 3 binary variables 001 and C by the pattern 010. Thus, the function that separates the mature miRNAs from the rest becomes f(V1, V2, V3) = sign( (not V1) **×** (not V2) **×** V3 + (not V1) **×** V2 **×** (not V3) – 0.5), where (not V1 ) is 1 if V1 is 0. This simplifies to f(V1, V2, V3) = sign((not V2) **×** V3 + V2 **×** (not V3) – 0.5) which is a non-linear decision surface due to the interaction terms (products) between the variables. Obviously, whether non-linearities will intrude depends on the exact binary encoding, i.e,. which binary patterns are associated to which nucleotides and the true classifying function. In contrast, the distributed encoding never introduces non-linearities for simple OR functions. The binary encoding suggested may also have the opposite effect, i.e., turn non-linearities to linearities. However, this will happen only in case the true classifying function is indeed non-linear.

### Overhangs calculation

The procedure for calculating the length of the overhangs is described for the k55 end and is similar for the k33 end. Based on the secondary structure of the hairpin, we identify the position of the base that matches k55 on the opposite strand, say k’55. The overhang length is obviously k33 – k’55. However, k55 does not always have a matching base. In this case, we move *x* positions to the left (inside the hairpin) until we find a base on the 5’ arm that has a matching base on the 3’ arm, say on position k’55. The overhang length is then computed as k33 – k’55 + *x*. Of course, when k55 has a matching base *x*=0 and the two computations coincide. The statistical distributions of overhangs’ length are shown below, Figure F1 in S1 Text.


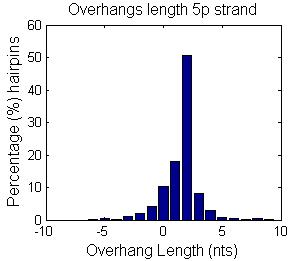

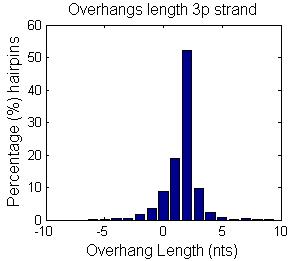


**Figure F1. Statistical distribution of the overhangs’ length.** The most frequent value is 2nts for both 5p and 3p strand miRNAs.

### Tip calculation

To calculate the tip we identify the last matching nucleotides before the tip, which correspond to the loop start and loop end position, respectively. If the tip is T and the last matching nucleotides are X and X’, then X < T < X’ and T = X + ceil ((X’-X) / 2), ceil refers to rounding toward positive infinity.

### All Corner Sum Absolute Error (ACSAE) – End Absolute Error

For example, if the true positions are *k55 =* XX_55_, *k53 =* XX_53_, *k35=* XX_35_ and *k33 =* XX_33_, and the predicted positions are YY_55_, YY_53_, YY_35_, YY_33_ respectively, then the ACSAE on this duplex is ACSAE = (|XX_55_ – YY_55_| + |XX_53_ – YY_53_| + |XX_35_ – YY_35_| + |XX_33_ – YY_33_|). The EAE for each duplex end is |XX_55_ – YY_55_|, |XX_53_ – YY_53_|, |XX_35_ – YY_35_|, |XX_33_ – YY_33_|, respectively.

### Set of features used

We tried several features before deciding which ones to use for our final model. During that process the degree of the kernel *d* and the cost parameter *c* of the SVM were set to d = 3 and c = 2, respectively and the flanking sequence length *l* (number of nucleotides before and after the duplex) to l = 13.

In the first model “thermodynamics”, we use only thermodynamics information to train and test our algorithm. Thermodynamics information was obtained by using “UNAFold.pl” program, <http://mfold.rna.albany.edu/?q=unafold-man-pages/UNAFold.pl>. “UNAFold.pl” program returns a file .det by which we extract this information.

In the second model “Sequence - Structure” we used sequence and structure information. To obtain the structure information we use RNAfold program with default parameters, (-p -d0 -noLP –noPS), and we keep the dot – bracket notation.

In the third model “Sequence - Entropy” we used sequence and entropy information. The entropy information was produced by using RNAfold’ utility: “mountain.pl”, <http://web.mit.edu/seven/src/ViennaRNA-1.5/Utils/>.

In the fourth model “Sequence”, only sequence information was used as discussed in the main text.

The mean prediction accuracies of these four models versus the All Corner Sum Absolute Error, (ACSAE, see above) are shown in Figure F2 in S1 Text. Based on these results we decide to optimize the two best models, namely “Sequence” and “Sequence - Entropy”. For both algorithms the degree of the kernel and the cost parameter of the SVM, and the flanking sequence length (number of nucleotides before and after the duplex) were optimized using five-fold cross validation. In both cases the best performing parameters were found to be: d = 3, c = 0.01 and l = 10. The mean prediction accuracies of the optimized models versus the ACSAE are shown in Figure F3 in S1 Text. Even though, the “Sequence – Entropy” model seems to have better performance than the “Sequence” model, the observed differences were not statistically significant and we decided to select the simplest model.


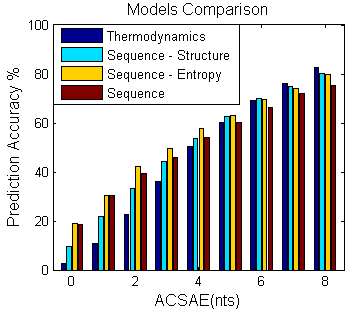


**Figure F2. Mean prediction accuracies.** The mean prediction accuracy shown in the figure was calculated in the following way. During each five-fold cross validation, 5 models were produced M1, M2, M3, M4, M5. For each model we calculate its prediction accuracy PA1, PA2, PA3, PA4 and PA5 and average all of them to obtain the mean prediction accuracy, MPA, MPA= (PA1 + PA2 + PA3 + PA4 + PA5)/ 5.


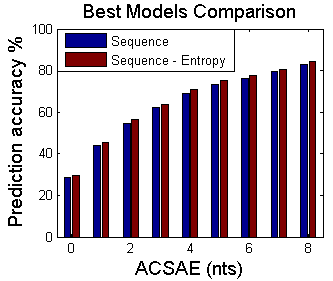


**Figure F3. Mean prediction accuracy for the “Sequence” and “Sequence - Entropy” models.** The prediction accuracy shown in the figure was calculated as in Figure F2 in S1 Text. Prediction accuracies were obtained using the best performing combination of the degree of the kernel d, the cost c and the flanking sequence length l. The “Sequence – Entropy” model has better performance than the “Sequence” model, however by performing Wilcoxon “ranksum” tests we note that the observed difference was not statistically significant.


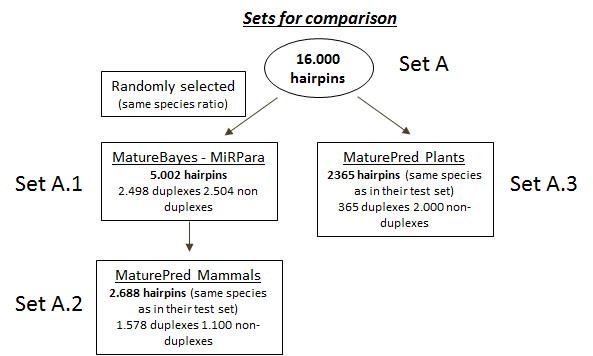


**Figure F4. Building test sets procedure.** Briefly, we start with all hairpins in miRBase version 19.0 and exclude the ones previously seen by any of the compared tools (during training or parameter optimization), resulting in ~16.000 hairpins. This set, hereby termed “Test Set A” is used to extract test sets for the various comparisons according to the specifications of each tool. Test Set A.1 was used to evaluate MatureBayes and MiRPara and was generated by randomly selecting ~5.000 hairpins, while maintaining the same species ratio of the original test set Α, out of which ~2.500 had known duplexes. Test Set A.2 consisted of hairpins (2688, 1578 with known duplexes) from Test Set A.1 which belonged to the species used in the original test set of MaturePred_Mammals. Comparison with MaturePred was also done for plants, using the Test Set A.3. This set consisted of all plant hairpins from Test Set A that belonged to the species used in the original test set of MaturePred_Plants (2365, 365 with known duplexes).

### Obtain other programs’ predictions

*MatureBayes* was the first tool specifically developed to address the problem of mature miRNA identification. It utilizes a Naive Bayes classifier to identify mature miRNA candidates based on sequence and secondary structure information of their miRNA precursors [[1](#_ENREF_1)]. It generates one prediction per strand and uses the 2nts overhang rule to define the predicted miRNA* on the opposite strand, creating two hypothetical duplexes. For this comparison, MiRduplexSVM was trained with the original training set of MatureBayes and both algorithms were evaluated on Test-Set A1. MatureBayes’ predictions were obtained using the downloadable version of the algorithm. The ACSAE was applied on MatureBayes’ hypothetical duplex which obtained the highest score. The EAE were in turn applied on the strand-specific molecules of the highest scoring candidate duplex for both tools.

*MiRPara* is another SVM-based, software tool for mature miRNA prediction [[2](#_ENREF_2)]. We compare MiRduplexSVM with the stand alone application, MiRPara 4.2, which was available at the time of evaluation. We trained our algorithm with all duplexes in miRBase version 13.0, which was used as a training set for MiRPara 4.2, and both algorithms were tested on Test Set A.1 (Figure F4, in S1 Text). MiRPara generates several independent predictions for each strand of any given hairpin, whereas MiRduplexSVM predicts only one duplex per hairpin. In order to compare the two tools in terms of the ACSAE we needed to produce a single hypothetical duplex for MiRPara. To this end, we consider the highest scoring predictions per strand as the two sides of the hypothetical duplex of any given hairpin. For comparisons using the EAE, we contrasted the top scoring prediction of MiRPara per strand against the prediction of MiRduplexSVM for the same strand. Strand-specific comparisons were performed independently of one another, i.e. there was no requirement that both strands produce a mature miRNA.

*MaturePred* [[3](#_ENREF_3)] employs an SVM classifier to predict the region, which is most likely to contain the mature miRNA molecule in each strand of a hairpin. It consists of two separate models, one specialized in plants, hereby termed MaturePred_Plants, which has been trained and tested on plant miRNAs and one specialized in mammals, hereby named MaturePred_Mammals, which has been trained and tested on mammalian miRNAs. We compare MiRduplexSVM with each model separately. In each case we train MiRduplexSVM with the respective MaturePred’s training set and evaluate performances on Test Sets A.2 (for mammals) and A.3 (for plants). In both comparisons, MaturePred’s predictions were acquired by using the online version of the respective model following the recommendations on its web site. As in the case of MiRPara, MaturePred gives multiple independent predictions per strand. Thus, we measure the ACSAE and EAE for each corner as described for MiRPara.

*MiRdup*[[4](#_ENREF_4)] is a state of the art method for the identification of the most likely miRNA location within a given pre-miRNA or the validation of a candidate miRNA. MiRdup is based on a random forest classifier trained with experimentally validated miRNAs from miRbase, with features that characterize the miRNA–miRNA* duplex. MiRdup predicts the most probable miRNA duplex on a given hairpin. Both MiRduplexSVM and MiRdup were trained on 70% of miRBase 17.0 (658 hairpins) and tested on the remaining 30% (290 hairpins). This was done since the MiRdup’s downloadable model was trained on the entire miRBase 19.0, and a lot of errors occurred when we tried to use it.

Table T1 Accurate predictions, up to 20 nts deviation

|  | All Corners Sum Absolute Error in nucleotides | | | | | | | | | | | | | | | | | | | | |
| --- | --- | --- | --- | --- | --- | --- | --- | --- | --- | --- | --- | --- | --- | --- | --- | --- | --- | --- | --- | --- | --- |
|  | <= 0 | <= 1 | <= 2 | <= 3 | <= 4 | <= 5 | <= 6 | <= 7 | <= 8 | <= 9 | <= 10 | <= 11 | <= 12 | <= 13 | <= 14 | <= 15 | <= 16 | <= 17 | <= 18 | <= 19 | <= 20 |
| MiRduplexSVM/ MatureBayes - Prediction accuacy % | 7.61%/ 0.08%  *** | 16.38%/ 0.44%  *** | 25.52%/ 1.25%  *** | 33.41%/ 4.59%  *** | 38.61%/ 9.86%  *** | 42.95%/ 17.07%  *** | 47.91%/ 28.1%  *** | 51.57%/ 36.19%  *** | 54.91%/ 42.47%  *** | 57.25%/ 47.91%  *** | 59.86%/ 54.15%  *** | 62.04%/ 57.29%  *** | 63.81%/ 61.27%  * | 65.66%/ 64.29%  ns | 67.11%/ 67.15%  ns | 68.72%/ 68.84%  ns | 70.21%/ 71.3%  ns | 71.46%/ 72.46%  ns | 72.95%/ 73.95%  ns | 73.95%/ 74.64%  ns | 74.96%/ 75.6%  ns |
| MiRduplexSVM/ MiRPara - Prediction accuacy % | 11.58%/ 0.21%  *** | 23.89%/ 1.95%  *** | 36.26%/ 6.58%  *** | 44.95%/ 15.79%  *** | 52.37%/ 26.47%  *** | 57.05%/ 38.95%  *** | 60.63%/ 48.68%  *** | 63.58%/ 56.47%  *** | 66.79%/ 63.11%  ** | 69.26%/ 68.11%  ns | 70.74%/ 72.11%  ns | 72.79%/ 74.58%  ns | 74.63%/ 76.32%  ns | 76.11%/ 78%  ns | 77.26%/ 79.16%  ns | 78.68%/ 80.16%  ns | 80.21%/ 81.16%  ns | 80.84%/ 82.05%  ns | 81.89%/ 83%  ns | 82.79%/ 83.74%  ns | 83.53%/ 84%  ns |
| MiRduplexSVM/ MaturePred_Plants - Prediction accuacy % | 28.77%/ 6.58%  *** | 40.82%/ 11.78%  *** | 46.58%/ 14.79%  *** | 48.49%/ 16.16%  *** | 50.14%/ 21.92%  *** | 52.05%/ 25.48%  *** | 53.7%/ 31.51%  *** | 54.25%/ 32.33%  *** | 57.81%/ 34.52%  *** | 58.63%/ 36.71%  *** | 59.45%/ 39.73%  *** | 59.73%/ 41.37%  *** | 61.64%/ 43.56%  *** | 63.56%/ 45.75%  *** | 64.93%/ 50.41%  *** | 65.75%/ 51.51%  *** | 67.67%/ 52.88%  *** | 67.67%/ 53.42%  *** | 68.49%/ 55.62%  *** | 69.32%/ 56.99%  *** | 69.86%/ 58.9%  ** |
| MiRduplexSVM/ MaturePred_Mammals - Prediction accuacy % | 11.85%/ 0.25%  *** | 26.93%/ 1.14%  *** | 40.87%/ 3.23%  *** | 51.08%/ 5.83%  *** | 58.3%/ 7.92%  *** | 63.31%/ 10.65%  *** | 68.19%/ 14.7%  *** | 71.61%/ 17.74%  *** | 75.22%/ 20.85%  *** | 77.19%/ 23.07%  *** | 79.66%/ 25.48%  *** | 81.18%/ 27.44%  *** | 82.83%/ 29.85%  *** | 84.41%/ 31.94%  *** | 85.61%/ 33.84%  *** | 86.76%/ 35.8%  *** | 87.45%/ 37.83%  *** | 88.21%/ 40.18%  *** | 88.97%/ 42.65%  *** | 89.92%/ 44.8%  *** | 90.49%/ 47.34%  *** |
| MiRduplexSVM/ MiRdup - prediction accuracy % | 31.38%/ 1.38%  *** | 47.59%/ 2.41%  *** | 56.9%/ 8.28%*** | 64.83%/  14.83%  *** | 73.1%/ 21.72%  *** | 75.52%/ 27.59%  *** | 78.97%/ 34.14%  *** | 81.72%/ 42.41%  *** | 83.45%/ 46.9%  *** | 86.55%/ 54.48%  *** | 88.62%/ 63.79%  *** | 88.97%/ 67.24%  *** | 90.69%/ 71.72%  *** | 91.38%/ 73.1%  *** | 91.72%/ 75.52%  *** | 92.76%/ 77.24%  *** | 93.79%/ 78.97%  *** | 94.14%/ 82.41%  *** | 94.14%/ 86.21%  *** | 94.48%/ 87.59%  ** | 95.86%/ 88.97%  ** |
| MiRduplexSVM/ Simple Geometric Locator - Prediction accuacy % | 31.38%/ 1.03%  *** | 47.59%/ 4.83%  *** | 56.9%/ 12.76%  *** | 64.83%/ 20%  *** | 73.1%/ 28.62%  *** | 75.52%/ 38.62%  *** | 78.97%/ 50.69%  *** | 81.72%/ 60.34%  *** | 83.45%/ 69.66%  *** | 86.55%/ 74.48%  *** | 88.62%/ 78.97%  ** | 88.97%/ 80.34%  ** | 90.69%/ 81.38%  *** | 91.38%/ 83.1%  ** | 91.72%/ 85.86%  * | 92.76%/ 88.28%  * | 93.79%/ 91.72%  ns | 94.14%/ 92.07%  ns | 94.14%/ 93.1%  ns | 94.48%/ 93.45%  ns | 95.86%/ 93.45%  ns |

The sum of the absolute error taken over all four ends of the predicted (MiRduplexSVM) or the hypothetical (MaturePredPlants, MaturePredMammals, MiRPara, MatureBayes) duplexes is calculated. MiRduplexSVM has been trained on each program’s training set and their performance has been accessed on a common blind test set. Fisher exact test were performed to examine if the observed differences are statistical significant. *** corresponds to pvalue ≤ 0.001, ** to pvalue ≤ 0.01, * to pvalue ≤ 0.05, and ns to non statistical.

Table T2 Accurate predictions, up to 8 nts deviation.

|  |  | End Absolute Error (EAE) | | | | | | | | |
| --- | --- | --- | --- | --- | --- | --- | --- | --- | --- | --- |
|  |  | <= 0 | <= 1 | <= 2 | <= 3 | <= 4 | <= 5 | <= 6 | <= 7 | <= 8 |
| MiRduplexSVM/ MatureBayes - Prediction accuracy % | k55 | 33.93%/ 7.79%  *** | 47.07%/ 28.34%  *** | 57.03%/ 51.19%  *** | 64.58%/ 63.68%  ns | 70.14%/ 70.58%  ns | 75.19%/ 74.48%  ns | 78.53%/ 77.44%  ns | 80.84%/ 79.61%  ns | 83.44%/ 81.35%  * |
|  | k53 | 22.72%/ 8.96%  *** | 42.75%/ 27.23%  *** | 55.46%/ 45.55%  *** | 63.46%/ 58.44%  *** | 69.65%/ 67.54%  * | 73.78%/ 73.13%  ns | 77.55%/ 76.74%  ns | 80.67%/ 79.72%  ns | 82.84%/ 81.27%  * |
|  | k35 | 32.15%/ 6.9%  *** | 46.85%/ 34.66%  *** | 56%/ 47.2%  *** | 64.23%/ 58.49%  *** | 70.73%/ 68.4%  * | 74.88%/ 74.85%  ns | 77.58%/ 78.85%  ns | 80.49%/ 80.75%  ns | 82.63%/ 82.63%  ns |
|  | k33 | 25.28%/ 10.66%  *** | 45.21%/ 32.89%  *** | 56.5%/ 49.68%  *** | 64.36%/ 60.47%  *** | 70.6%/ 69.62%  ns | 74.8%/ 75.12%  ns | 77.71%/ 79.16%  ns | 80.22%/ 81.31%  ns | 83.1%/ 83.18%  ns |
| MiRduplexSVM/ MiRPara - Prediction accuracy % | k55 | 44.34%/ 14.03%  *** | 58.2%/ 40.65%  *** | 66.45%/ 61.68%  *** | 72.75%/ 73.44%  ns | 77.86%/ 78.59%  ns | 81.07%/ 80.93%  ns | 83.95%/ 82.6%  ns | 85.62%/ 84.79%  ns | 87.54%/ 85.66%  * |
|  | k53 | 28.99%/ 16.05%  *** | 51.97%/ 42.15%  *** | 65.23%/ 60.81%  *** | 71.91%/ 72.26%  ns | 77.2%/ 77.62%  ns | 80.75%/ 81.17%  ns | 83.85%/ 82.81%  ns | 85.49%/ 83.99%  ns | 87.16%/ 85.03%  * |
|  | k35 | 43.43%/ 20.95%  *** | 58.9%/ 52.3%  *** | 68.08%/ 67.98%  ns | 73.6%/ 75.32%  ns | 78.44%/ 79.55%  ns | 81.52%/ 81.72%  ns | 83.65%/ 83.38%  ns | 85.14%/ 84.77%  ns | 87.14%/ 86.15%  ns |
|  | k33 | 31.75%/ 14.93%  *** | 54.71%/ 40.72%  *** | 66.22%/ 61.37%  *** | 73.32%/ 73.73%  ns | 78.3%/ 79.18%  ns | 81.65%/ 82.36%  ns | 84.12%/ 84.5%  ns | 85.65%/ 85.78%  ns | 87.31%/ 86.97%  ns |
| MiRduplexSVM/ MaturePred_Plants - Prediction accuracy % | k55 | 37.14%/ 39.62%  ns | 46.83%/ 50.69%  * | 52.22%/ 57.47%  ** | 56.23%/ 62.71%  *** | 60.89%/ 68.32%  *** | 64.02%/ 71.01%  *** | 66.86%/ 74.58%  *** | 69.85%/ 76.84%  *** | 72.1%/ 78.88%  *** |
|  | k53 | 34.09%/ 30.95%  * | 45.88%/ 49.96%  * | 52.37%/ 56.74%  * | 55.72%/ 62.2%  *** | 60.16%/ 67.52%  *** | 64.31%/ 70.21%  *** | 66.28%/ 73.71%  *** | 70.07%/ 77.2%  *** | 72.54%/ 78.51%  *** |
|  | k35 | 37.73%/ 40.68%  ns | 46.13%/ 48.05%  ns | 52.03%/ 55.2%  ns | 57.48%/ 61.97%  ** | 61.39%/ 66.4%  ** | 65.44%/ 70.52%  ** | 69.57%/ 73.69%  ** | 72.37%/ 76.49%  ** | 74.72%/ 78.78%  ** |
|  | k33 | 33.24%/ 33.9%  ns | 46.2%/ 46.79%  ns | 52.32%/ 53.87%  ns | 58.07%/ 61.75%  * | 62.64%/ 66.54%  * | 66.47%/ 70.08%  * | 69.2%/ 73.25%  * | 72.37%/ 76.27%  * | 75.31%/ 79%  * |
| MiRduplexSVM/ MaturePred_Mammals - Prediction accuracy % | k55 | 50.69%/ 13.44%  *** | 67.03%/ 25.94%  *** | 75.96%/ 37.62%  *** | 82.61%/ 46.94%  *** | 86.79%/ 56.1%  *** | 89.69%/ 64.47%  *** | 92.11%/ 69.69%  *** | 93.35%/ 74.11%  *** | 94.77%/ 80.33%  *** |
|  | k53 | 31.54%/ 8.31%  *** | 56.44%/ 22%  *** | 71.83%/ 35.11%  *** | 80.19%/ 45.13%  *** | 85.65%/ 55.49%  *** | 89.03%/ 63.66%  *** | 91.31%/ 69.12%  *** | 93.02%/ 74.16%  *** | 94.54%/ 80.1%  *** |
|  | k35 | 48.56%/ 16.03%  *** | 66.36%/ 25.3%  *** | 76.14%/ 33.23%  *** | 83.09%/ 42.96%  *** | 87.21%/ 48.33%  *** | 90.22%/ 53.29%  *** | 92.12%/ 57.78%  *** | 93.74%/ 62.23%  *** | 95%/ 66.91%  *** |
|  | k33 | 39.67%/ 16.4%  *** | 63.11%/ 28.64%  *** | 75.72%/ 36.61%  *** | 82.62%/ 43.56%  *** | 86.61%/ 49.95%  *** | 90.13%/ 54.36%  *** | 92.68%/ 59.64%  *** | 93.74%/ 64.32%  *** | 95.04%/ 69.23%  *** |
| MiRduplexSVM/ MiRdup - Prediction accuracy % | k55 | 65.17%/ 15.86%  *** | 79.31%/ 39.31%  *** | 86.55%/ 57.24%  *** | 90%/ 75.86%  *** | 92.76%/ 84.14%  *** | 94.48%/ 89.66%  * | 96.55%/ 92.76%  * | 97.93%/ 95.52%  ns | 98.62%/ 97.24%  ns |
|  | k53 | 51.38%/ 14.14%  *** | 76.21%/ 42.41%  *** | 83.45%/ 62.76%  *** | 89.31%/ 72.41%  *** | 92.07%/ 79.31%  *** | 94.83%/ 87.59%  ** | 97.59%/ 92.07%  ** | 98.28%/ 94.48%  * | 98.97%/ 96.9%  ns |
|  | k35 | 56.9%/ 16.9%  *** | 77.93%/ 37.24%  *** | 83.79%/ 56.9%  *** | 90%/ 70.69%  *** | 94.48%/ 83.79%  *** | 95.52%/ 89.66%  ** | 96.21%/ 92.41%  * | 97.59%/ 94.83%  ns | 97.59%/ 95.52%  ns |
|  | k33 | 57.59%/ 14.14%  *** | 76.55%/ 33.1%  *** | 85.17%/ 52.76%  *** | 90.34%/ 66.9%  *** | 93.1%/ 81.38%  *** | 96.55%/ 88.97%  *** | 96.55%/ 91.38%  ** | 97.59%/ 94.83%  ns | 97.93%/ 97.24%  ns |
| MiRduplexSVM/ Simple Geometric Locator - Prediction accuracy % | k55 | 65.17%/ 15.52%  *** | 79.31%/ 44.83%  *** | 86.55%/ 73.45%  *** | 90%/ 84.48%  * | 92.76%/ 92.07%  ns | 94.48%/ 95.17%  ns | 96.55%/ 96.21%  ns | 97.93%/ 97.59%  ns | 98.62%/ 97.59%  ns |
|  | k53 | 51.38%/ 13.45%  *** | 76.21%/ 43.45%  *** | 83.45%/ 70.34%  *** | 89.31%/ 83.79%  * | 92.07%/ 91.03%  ns | 94.83%/ 93.79%  ns | 97.59%/ 95.52%  ns | 98.28%/ 97.93%  ns | 98.97%/ 97.93%  ns |
|  | k35 | 56.9%/ 16.55%  *** | 77.93%/ 50.69%  *** | 83.79%/ 74.83%  ** | 90%/ 85.17%  ns | 94.48%/ 91.72%  ns | 95.52%/ 93.45%  ns | 96.21%/ 94.83%  ns | 97.59%/ 95.86%  ns | 97.59%/ 97.24%  ns |
|  | k33 | 57.59%/ 18.28%  *** | 76.55%/ 50.34%  *** | 85.17%/ 73.79%  *** | 90.34%/ 85.17%  * | 93.1%/ 90.69%  ns | 96.55%/ 93.45%  ns | 96.55%/ 94.48%  ns | 97.59%/ 96.21%  ns | 97.93%/ 97.59%  ns |

The absolute error for each one of the four ends of the duplex is calculated independently. MiRduplexSVM has been trained on each program’s training set and their performance has been accessed on a common blind test set. Fisher exact test were performed to examine if the observed differences are statistical significant. *** corresponds to pvalue ≤ 0.001, ** to pvalue ≤ 0.01, * to pvalue ≤ 0.05 and ns to non statistical.

1. Gkirtzou K, Tsamardinos I, Tsakalides P, Poirazi P (2010) MatureBayes: a probabilistic algorithm for identifying the mature miRNA within novel precursors. PLoS One 5: e11843.

2. Wu Y, Wei B, Liu H, Li T, Rayner S (2011) MiRPara: a SVM-based software tool for prediction of most probable microRNA coding regions in genome scale sequences. BMC Bioinformatics 12: 107.

3. Xuan P, Guo M, Huang Y, Li W, Huang Y (2011) MaturePred: efficient identification of microRNAs within novel plant pre-miRNAs. PLoS One 6: e27422.

4. Leclercq M, Diallo AB, Blanchette M (2013) Computational prediction of the localization of microRNAs within their pre-miRNA. Nucleic Acids Res.
